# Using partially ordered sets to represent and predict true patterns of gene response to treatments

**DOI:** 10.1186/1471-2105-14-S17-A20

**Published:** 2013-10-22

**Authors:** Nam S Vo, Vinhthuy Phan

**Affiliations:** 1 Department of Computer Science, The University of Memphis, Memphis, TN 38152, USA

## Background

Advances in biotechnology have empowered high-throughput measurement of gene expression levels for tens of thousands of genes simultaneously. This means that one sample size must be used for all genes in most experimental designs [1,2], which implies that patterns of response of highly variantly expressed genes might not be measured accurately. Response patterns of gene expression data with multiple treatments have been characterized using *post hoc* pairwise comparisons by several researchers [3,4]. Nevertheless, these researchers did not address how to cope with highly variantly expressed genes with inaccurate patterns due to having too few experimental samples.

## Results

We show that dependencies of pairwise comparison outcomes in *post hoc* calculations can be exploited to infer true response patterns of genes with inaccurate patterns due to having too few experimental samples.

Characterizing such response patterns as partially ordered sets, we show that *linearly orderable* patterns are more likely true patterns and those that are not *linearly orderable* cannot be true patterns. We propose a strategy to predict most likely linearly orderable extensions of such patterns. Using microarray data of rats' liver cells, we showed that this approach yielded more and better functionally enriched gene lists than a conventional approach.

## Conclusions

This approach opens up opportunities to design cost-effective experiments, in which only a conservatively large sample size is needed to collect expression levels of almost all genes. For most genes, such a sample size is sufficient. For highly variantly expressed genes, our method can help infer true response patterns.
